# Air pollution influences the incidence of otitis media in children: A national population-based study

**DOI:** 10.1371/journal.pone.0199296

**Published:** 2018-06-28

**Authors:** Mina Park, Jiyeon Han, Myoung-jin Jang, Myung-Whan Suh, Jun Ho Lee, Seung Ha Oh, Moo Kyun Park

**Affiliations:** 1 Department of Otorhinolaryngology-Head and Neck Surgery, Seoul Medical Center, Seoul, South Korea; 2 Medical Research Collaborating Center, Seoul National University Hospital, Seoul, South Korea; 3 Department of Otorhinolaryngology-Head and Neck Surgery, Seoul National University College of Medicine, Seoul National University Hospital, Seoul, South Korea; 4 Sensory Organ Research Institute, Seoul National University Medical Research Center, Seoul, South Korea; Telethon Institute for Child Health Research, AUSTRALIA

## Abstract

**Background:**

Otitis media (OM) is a major reason for children’s visits to physicians and a major cause of their being treated with antibiotics. It not only causes economic burdens but also influences hearing, speech, and education. To our knowledge, no nationwide population-based study has assessed the association between air pollution and OM. Therefore, this study evaluated the association between air pollution levels and the incidence of OM.

**Methods:**

We identified cases of OM that occurred in South Korea between January 2011 and December 2012 from the Korea National Health Insurance Service-National Sample Cohort database, and evaluated its relationship with five air pollutants: particulate matter (PM_10_, particulates ≤10 μm in diameter), nitrogen dioxide (NO_2_), ozone (O_3_), sulfur dioxide, and carbon monoxide. Associations between the weekly incidence of OM and the five air pollutants were analyzed using generalized estimating equations. Conditional logistic regression analysis was used to obtain odds ratios (ORs) and their 99.9% Bonferroni-corrected confidence intervals after adjusting for gender, age, season, and region.

**Results:**

We based our analysis on 160,875 hospital visits for OM by children aged <15 years. Correlations with higher concentrations of the five pollutants showed higher ORs than did the reference values at most time lags. PM_10_ had the largest influence on the OM incidence at a time lag of 0 weeks, whereas NO_2_ and O_3_ had the largest impacts on OM incidence at time lags of 1 and 4 weeks, respectively.

**Conclusion:**

These findings support the notion that the incidence of OM is associated with ambient air pollution.

## Introduction

Otitis media (OM) is a major reason for children’s visits to physicians [1] and a major cause of their being treated with antibiotics [2]. Moreover, OM has effects on hearing, speech, and educational performance. The total economic burden of acute OM is estimated to be approximately $606 million per year in South Korea. Moreover, the average cost of admission for acute OM per person was about $1,690 per year, and that of outpatient visits was $199 per person [3].

OM is a common disease in children younger than 10 years, and it affects about 80% of all children at some point [4]. OM is an immune response to a microbial disease, typically an upper respiratory infection, especially in children [5]. The effects of meteorological (i.e., temperature and air humidity) and environmental (i.e., passive smoking and air pollution) factors on OM have also been studied [6–13].

Several European and American epidemiological studies have shown relationships between air pollution and OM in children. In 1993, Sperm and Branica showed the correlation between monthly temperature/air humidity and OM. The highest incidence of OM was in winter and the lowest was in summer [13]. In a cross-sectional study of 393 children in three different areas of São Paulo, Brazil, examining ambient air pollution, Ribeiro and Cardoso [14] found a positive association between air pollutant levels and the prevalence of respiratory symptoms, including ear infections. Caceres Udina *et al*. [10] conducted a prospective cohort study of 229 newborns in Spain and showed that the incidence of acute OM was high in infants living in a polluted area. Brauer *et al*. [9] studied two prospective birth cohorts of about 4,000 infants aged 0–2 years in the Netherlands and Germany and reported that OM occurred in proportion to traffic-related air pollutants. Zemek *et al*. [7] published data from a case-crossover study of about 14,000 children aged 1–3 years in Canada that showed an association of ambient air pollutants with emergency room visits for OM. Deng *et al*. [11] conducted a prospective cohort study of 1,617 preschool-age children in China and found that exposure to an industrial air pollutant (SO_2_) was associated with the onset of OM.

However, these studies investigated specific regions or populations. To our knowledge, no study has used nationwide data to investigate the association between ambient air pollutants and OM. South Korea would be a good place to study the effect of air pollution on disease at a national scale because it has a mandatory national health care system that includes everyone and a well-established air pollution monitoring system that covers the entire country [15–17]. In addition, the daily air quality index of South Korea shows that the air pollutant levels are typically 2–5 times higher than those deemed safe by the World Health Organization [18].

Therefore, in this study, we evaluated the relationship between air pollution and the incidence of OM based on a nationwide representative sample of 1 million South Koreans.

## Methods

### Database

South Korea has a distinctive health insurance system in that all people are compulsorily enrolled in the Korean National Health Insurance Service (KNHIS), established by the government to provide affordable health coverage for the whole population at low cost. Because all Koreans are given a unique identification number at birth, healthcare records of individuals do not overlap and are not omitted. Thus, the KNHIS maintains comprehensive data on the healthcare of individuals that includes information on diagnostic codes, procedures, and prescriptions [19].

### Study sample

The KNHIS constructed a National Sample Cohort (NSC) in 2002, comprising 1 million nationally representative members, ~2% of the entire population of 50 million. This data set includes all medical claims during the study period and patient information [sex, age group (0, 1–4, 5–9, 10–14, …, 80–84, and ≥85 years), and residence area] [20]. The Seoul National University Hospital Institutional Review Board (1509-056-702) approved this study.

### Otitis media

All children aged <15 years (160,875 children) were tracked based on the index dates of visits to hospitals in January 2011 and December 2012 to identify children who visited clinics or hospitals for OM based on the International Classification of Disease, 10^th^ edition (ICD-10) codes H65.0 (acute serous OM), H65.1 (other acute nonsuppurative OM), H65.4 (other chronic nonsuppurative OM), H65.1 (other acute nonsuppurative OM), H65.1 (other acute nonsuppurative OM), H66 (suppurative and unspecified OM), H67.8 (OM in other diseases classified elsewhere), and H67.1 (OM in viral diseases classified elsewhere) [21].

### Air pollution

We obtained regional air pollution data from the Korean Ministry of the Environment for 2011–2012 (e-mail contact: airkorea@keco.or.kr), which were released in 2015. Air pollutants are measured continuously and automatically using monitoring devices that follow electrochemical theory. Acquired data are collected in real time by the National Ambient Air Monitoring Information System (NAMIS) and provided to the public through Air Korea in various forms (http://www.airkorea.or.kr/eng/information/publications_List). There are 313 monitoring stations that cover 79 areas in 16 administrative regions. All data from the 313 stations undergo two data screening steps. First, a computer program determines whether the data are abnormal only when information on the condition of the measurement equipment (e.g., calibration, inspection, or malfunction is available. Second, it regards the collected data as abnormal when the data exceed the normal range [22].

In the 16 administrative regions, five widely used air pollutant variables were assessed [16, 17]: particulate matter (PM_10_, particulates <10 μm in diameter, by a β-ray absorption method); nitrogen dioxide (NO_2_, by chemiluminescence); ozone (O_3_, by an ultraviolet photometric method); sulfur dioxide (SO_2_, by a pulse ultraviolet fluorescence method); and carbon monoxide (CO, by a non-dispersive infrared method). The values for all pollutants were originally provided on an hourly basis; we converted them to a weekly basis for the analyses. The values of each air pollutant are the averages of the concentrations measured at the monitoring stations in each administrative region. The median Pearson correlations of weekly mean air pollutants concentrations at different monitoring stations within a single administrative region were 0.92 (range 0.39–1) μg/m^3^ for PM_10_, 0.75 (range -0.23–1) ppm for NO_2_, 0.90 (range -0.23–1) ppm for O_3_, 0.74 (range -0.58–1) ppm for SO_2_, and 0.77 (range -0.36–1) ppm for CO.

To classify the concentration of each air pollutant, we followed the classification used by Air Korea (http://www.airkorea.or.kr/eng/cai/cai1), in which the concentration of each air pollutant is divided into four groups according to the comprehensive air-quality index. This is a way of describing ambient air quality based on the health risk of the air pollution [23].

### Statistical analysis

Any associations between concentrations of air pollutants and the incidence of OM were analyzed using generalized estimating equations (GEE) for panel data. An autoregressive working correlation matrix was used to account for correlations among repeated measures on the same subject. The lagged exposures of each air pollutant, up to 4 weeks (from time lag 0 to time lag 4 weeks), were examined and adjusted for sex, age (<5/≥5 years), season (summer/not summer), and region (16 administrative regions).

We used a weekly lag-range system for two reasons. First, OM does not cause acute symptoms such as fever and otalgia; it causes no symptoms or delayed hearing loss. Second, there are time intervals between air pollutant exposure, the occurrence of OM, and visiting a hospital. Because we derived the OM incidence from visits to clinics or hospitals, not emergency departments, we set the lag-range at week intervals.

The linearity assumption for logistic regression was checked by categorizing each air pollutant into multiple dichotomous variables of equal intervals and plotting the coefficient against the midpoints of each dichotomous variable; the linearity assumptions were not met. Concentrations of each air pollutant were categorized into four groups, considering the Korean National Standards for Air Quality (KNSAQ) and the maximum values. PM_10_ concentrations were grouped into good (0–30), moderate (31–80), unhealthy (81–150), and very unhealthy (≥151 μg/m^3^) according to KNSAQ. NO_2_ and O_3_ showing levels lower than the upper limit of moderate air quality were grouped into lower good (0–0.015), upper good (0.016–0.030), lower moderate (0.031–0.045), and upper moderate (≥0.046 ppm) using the good air quality criteria (0–0.03 ppm) from KNSAQ and equal group intervals. SO_2_ and CO showing levels lower than the upper limit of good air quality according to KNSAQ were grouped as quartiles, 0–0.00369, 0.00370–0.00479, 0.00480–0.00629, and ≥0.0063 ppm for SO_2_ and 0–0.419, 0.420–0.499, 0.500–0.589, and ≥0.590 ppm for CO (http://www.airkorea.or.kr/eng/cai/cai1). The first group (lowest concentration) was used as the reference exposure category in each case.

*P*-values <0.00067 (0.05/75) and Bonferroni-corrected confidence intervals (CIs) of the odds ratio (OR) and 99.9% CI were considered for statistical significance using the Bonferroni correction for 75 multiple tests (three group comparisons × five air pollutants × five lag times). All statistical analyses were performed using SAS (ver. 9.2; SAS Institute, Cary, NC, USA).

## Results

### Trends in OM and air pollution levels

**Fig 1:**
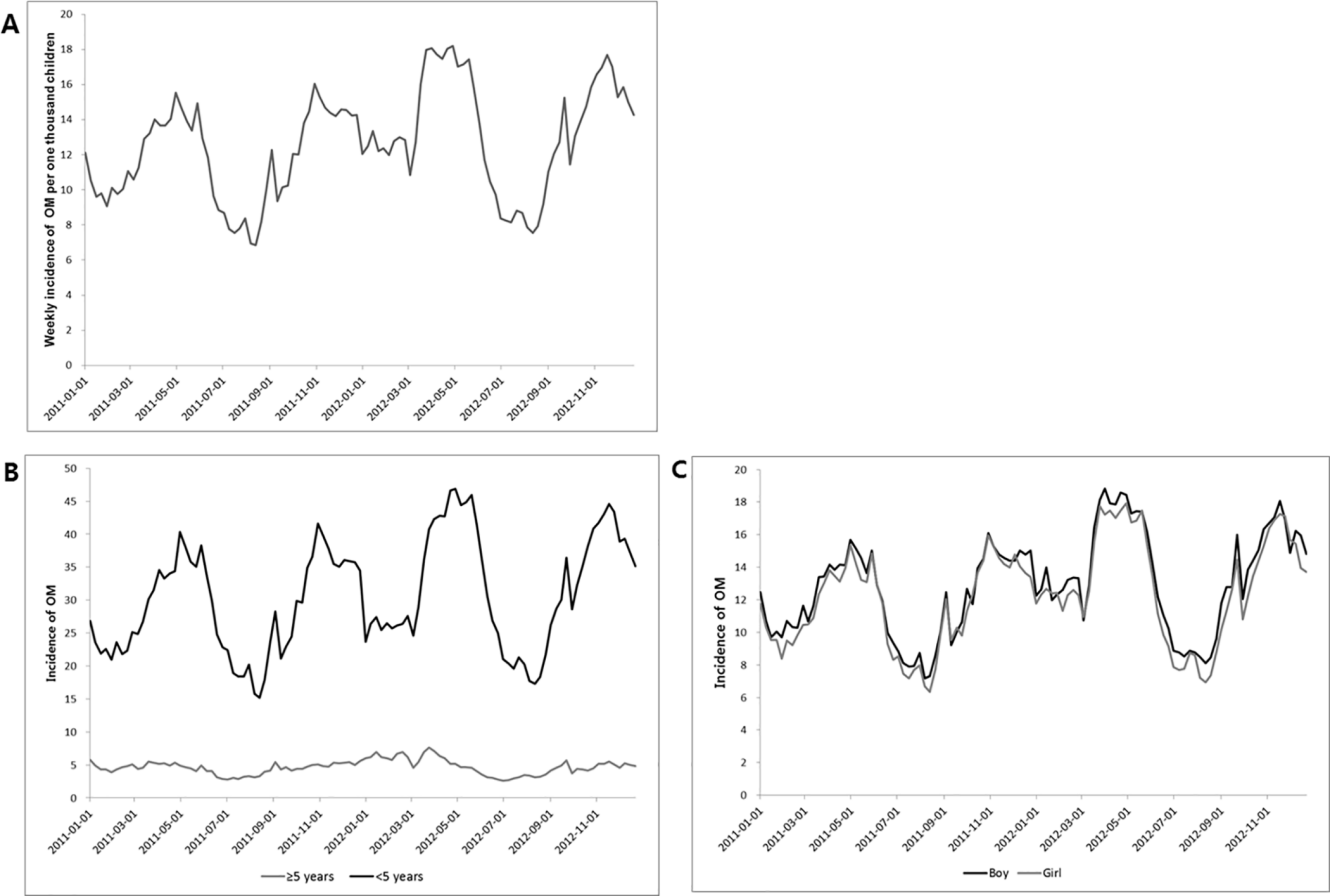
**Trends in weekly national otitis media (OM) cases per 1000 children in South Korea in total (A), by age (B), and by sex (C).** In 2011 and 2012, the weekly incidence of OM remained steady with seasonal variation, and the average was 12.6 cases per 1000 children. For age and sex, it was much higher in children <5 years, and comparable or somewhat higher in boys than in girls.

Over the 2 years of the study (2011–2012), the weekly national OM incidence increased from 11.7 in 2011 to 13.4 in 2012 per 1,000 children (OR = 1.14, 95% CI = 1.12–1.16; S1 Table) and showed seasonal variation, with troughs in the summer (spring compared to summer, OR = 1.52, 95% CI = 1.50–1.55; autumn to summer, OR = 1.43, 95% CI = 1.41–1.46; winter to summer, OR = 1.34, 95% CI = 1.31–1.36) (Fig 1A). Regarding age and gender, it was much higher in children < 5 years than in children ≥ 5 years (30.5 vs. 4. 7, respectively; OR = 0.15, 95% CI = 0.15–0.16; Fig 1B) with similar or slightly higher incidence in boys than in girls (12.9 vs. 12.2, respectively; OR = 1.05, 95% CI = 1.02–1.09; Fig 1C). The weekly OM incidence varied considerably by region (range 8.2 in Gangwon Province to 14.3 in Gyeonggi and Jeollanam Provinces; S2 Table).

The tendencies in nationwide weekly averages for each air pollutant by period are illustrated in S1 Fig. The median levels of weekly air pollutants during the study period were 42.7 (range 11.6–201.0) μg/m^3^ for PM_10_, 0.019 (range 0.004–0.058) ppm for NO_2_, 0.025 (range 0.007–0.06) ppm for O_3_, 0.005 (range 0.001–0.014) ppm for SO_2_, and 0.468 (range 0.201–1.227) ppm for CO. The pollutant levels were not correlated with one another during this period. The weekly concentrations of air pollutants did not show yearly trends, except for PM_10_, the concentration of which was higher in 2011 than in 2012 (median 53.2 vs. 48.2 μg/m^3^, respectively). Concerning seasonal variations, the levels of PM_10_ and O_3_ were highest in spring, whereas those of the other five pollutants were highest in winter (S1 Table).

### Association between OM, time lags, and air pollutant concentrations

Regarding PM_10_, the second and third levels of PM_10_ (moderate and unhealthy) were associated with OM incidence at all time lags and at time lags of 0, 1, and 3 weeks when compared with the first (good) group. The largest effects at the second and third levels were during the week of OM incidence (lag 0; OR = 1.09, 99.9% CI = 1.07–1.12 and OR = 1.06, 99.9% CI = 1.01–1.1, respectively) and at a time lag of 3 weeks (lag 3; OR = 1.09, 99.9% CI = 1.06–1.11 and OR = 1.06, 99.9% CI = 1.01–1.10, respectively). OM incidence was associated with the fourth (very unhealthy) PM_10_ level only during the week of OM incidence (OR = 1.34, 99.9% CI = 1.17–1.54), compared with the first (good) level of PM_10_, after adjusting for covariates.

In the case of NO_2_, the OM incidence was associated with the highest NO_2_ concentrations at all time lags, except for the 3-week time lag, and with the second- and third-highest levels, except for time lag 4. Regardless of the level, the largest effect was seen at time lag 1 (OR = 1.09, 99.9% CI = 1.05–1.12 for the second group, OR = 1.12, 99.9% CI = 1.08–1.16 for the third group, and OR = 1.15, 99.9% CI = 1.09–1.22 for the fourth group).

For O_3_, OM incidence was associated with the fourth highest level of O_3_ concentration at time lags 2–4, with the third highest levels at all time lags, and with the second highest levels at time lag 4. Regardless of the level, the largest magnitude of effect was shown at time lag 4 (OR = 1.05, 99.9% CI = 1.03–1.08 for the second highest level, OR = 1.08, 99.9% CI = 1.05–1.12 for the third, and OR = 1.16, 99.9% CI = 1.07–1.25 for the fourth level).

In the case of SO_2_, the three upper concentration quartiles showed higher risks of OM incidence than the lowest quartile did for most time lags. The effects of the three upper quartiles on OM were similar, with the largest effects at time lag 0 (OR = 1.08, 99.9% CI = 1.05–1.11 for the second, OR = 1.1, 99.9% CI = 1.07–1.13 for the third, and OR = e1.08, 95% CI = 1.05–1.12 for the fourth quartile).

Regarding CO, the three highest quartiles showed higher risks than the lowest quartile did at most time lags. The timing of the largest effects differed among the time lags (OR = 1.08, 99.9% CI = 1.06–1.11 at time lag 2 for the second; OR = 1.11, 99.9% CI = 1.08–1.14 at time lag 3 for the third; OR = 1.08, 99.9% CI = 1.05–1.12 at time lag 1 for the fourth quartile; Table 1).

**Table 1:** Adjusted association between exposure to ambient air pollutants and the incidences of otitis media in a nationwide population study for children in South Korea.

	Level	Lag 0		Lag 1		Lag 2		Lag 3		Lag 4	
		OR	99.9% CI*	OR	99.9% CI*	OR	99.9% CI*	OR	99.9% CI*	OR	99.9% CI*
**PM**_**10**_ (μg/m^3^)	0–30	1.00		1.00		1.00		1.00		1.00	
	31–80	1.09	[1.07, 1.12]	1.07	[1.05, 1.09]	1.04	[1.01, 1.06]	1.09	[1.06, 1.11]	1.03	[1.00, 1.05]
	81–150	1.06	[1.01, 1.10]	1.05	[1.01, 1.09]	1.01	[0.97, 1.05]	1.06	[1.01, 1.10]	0.98	[0.94, 1.02]
	≥151	1.34	[1.17, 1.54]	0.98	[0.83, 1.16]	1.01	[0.87, 1.17]	0.85	[0.72, 1.01]	1.11	[0.96, 1.30]
**NO**_**2**_ (ppm)	0–0.015	1.00		1.00		1.00		1.00		1.00	
	0.016–0.030	1.06	[1.03, 1.10]	1.09	[1.05, 1.12]	1.06	[1.03, 1.09]	1.07	[1.04, 1.10]	1.01	[0.99, 1.04]
	0.031–0.045	1.11	[1.07, 1.15]	1.12	[1.08, 1.16]	1.09	[1.06, 1.13]	1.08	[1.04, 1.12]	1.02	[0.98, 1.06]
	≥ 0.046	1.11	[1.05, 1.17]	1.15	[1.09, 1.22]	1.08	[1.02, 1.14]	1.05	[0.99, 1.10]	1.05	[1.00, 1.11]
**O**_**3**_ (ppm)	0–0.015	1.00		1.00		1.00		1.00		1.00	
	0.016–0.030	0.97	[0.95, 1.00]	0.98	[0.95, 1.00]	1.01	[0.98, 1.03]	1.00	[0.98, 1.03]	1.05	[1.03, 1.08]
	0.031–0.045	1.07	[1.03, 1.10]	1.05	[1.02, 1.08]	1.07	[1.03, 1.10]	1.07	[1.04, 1.11]	1.08	[1.05, 1.12]
	≥ 0.046	1.06	[0.99, 1.14]	1.07	[0.99, 1.15]	1.12	[1.04, 1.20]	1.11	[1.03, 1.20]	1.16	[1.07, 1.25]
**SO**_**2**_ (ppm)	0–0.00369	1.00		1.00		1.00		1.00		1.00	
	0.0037–0.00479	1.08	[1.05, 1.11]	1.05	[1.02, 1.07]	1.06	[1.03, 1.08]	1.09	[1.06, 1.12]	1.03	[1.01, 1.06]
	0.0048–0.00629	1.10	[1.07, 1.13]	1.08	[1.05, 1.11]	1.06	[1.03, 1.09]	1.08	[1.05, 1.11]	1.03	[1.00, 1.06]
	≥ 0.0063	1.08	[1.05, 1.12]	1.07	[1.03, 1.10]	1.01	[0.97, 1.04]	1.03	[0.99, 1.06]	0.99	[0.95, 1.02]
**CO** (ppm)	0–0.419	1.00		1.00		1.00		1.00		1.00	
	0.42–0.499	1.05	[1.02, 1.07]	1.07	[1.05, 1.10]	1.08	[1.06, 1.11]	1.06	[1.04, 1.09]	1.04	[1.01, 1.06]
	0.50–0.589	1.05	[1.03, 1.08]	1.08	[1.05, 1.11]	1.10	[1.07, 1.13]	1.11	[1.08, 1.14]	1.06	[1.03, 1.09]
	≥ 0.59	1.05	[1.01, 1.08]	1.08	[1.05, 1.12]	1.06	[1.03, 1.10]	1.06	[1.03, 1.10]	1.01	[0.98, 1.05]

The odd ratios (ORs) were adjusted for gender, age, season, and region.

*99.9% confidence intervals (CIs) were Bonferroni-corrected for multiple tests

Lag 0, 1, 2, 3, and 4 means the weeks prior to otitis media.

Regardless of the air pollutant and time lag, PM_10_ showed the greatest effect on OM incidence (OR, 1.34; 99.9% CI = 1.17–1.54) at time lag 0 and at concentrations ≥151. At the lag times when NO_2_ and O_3_ each showed the highest risk for affecting OM incidence (NO_2_, OR = 1.15, time lag 1; O_3,_ OR = 1.16, time lag 4), higher concentrations were associated with a higher risk of OM occurrence. PM_10_ was the single factor with the largest influence on OM incidence at time lag 0, whereas NO_2_ and O_3_ had the largest impacts on OM incidence at time lags 1 and 4, respectively (Table 1).

## Discussion

By analyzing the incidence of OM over the whole of South Korea in 2011 and 2012, we showed a significant relationship between ambient air pollutants and the incidence of OM in children aged <15 years. Among the five pollutants analyzed, PM_10_, NO_2_, and O_3_ showed the greatest effects at time lags 0, 1, and 4, respectively. Higher SO_2_ and CO levels showed increased influence on OM compared with the lowest concentration quartiles. Above all, PM_10_ was most strongly associated with OM. PM_10_ concentrations ≥151 μg/m^3^ showed a significantly greater impact on OM compared with PM_10_ ≤30 μg/m^3^ at time lag 0 (OR = 1.34, 99.9% CI = 1.17–1.54).

The associations between various air pollutants and OM were not always consistent. Regarding PM_10_, although there was a robust association between PM_10_ and OM, no association was detected in studies in Canada and China [7, 11]. In the present work and the Canadian study, OM was associated with NO_2_ [7], but in the Chinese study it was not [11]. The Canadian study concluded that interquartile range rises in SO_2_ levels were not correlated with emergency department visits due to OM [7]. Our study also found no linear association between the SO_2_ level and OM incidence. A German study concluded that the prevalence of OM was related to the SO_2_ level [24]. A Brazilian cross-sectional study of 11–13-year-old children found a positive relationship between the SO_2_ level and the prevalence of OM [14]. The Chinese study reported an association between SO_2_ and the incidence of OM [11].

Recent studies have explored the delayed effects of several air pollutants on the onset of OM based on daily or hourly approaches. In 2010, Zemek *et al*. [7] reported that the interquartile range increases in CO and NO_2_ were associated with emergency room visits for OM in 1–3-year-old children with a 2-day lag all year. In 2016, Kousha and Castner [25] showed that emergency department visits for OM increased 6–7 days and 3–4 days after exposure to increased O_3_ and PM, respectively. This suggests that the multipollutant Air Quality Health Index was closely associated with emergency department visits for OM. In 2017, Gestro *et al*. [26] used dose-response models separately on daily and hourly bases to demonstrate the association between temperature and the relative risk of OM, in which low temperature increases the risk OM.

We used the National Health Insurance Service-National Sample Cohort (NHIS-NSC) database, which covers a representative population-based cohort that is also nationwide in scope and large in scale, to overcome the weaknesses of cross-sectional data. Since those data were provided to the public in July 2014, over 100 studies have been published across broad cross-disciplinary fields using the NHIS-NSC database. In 2016, Lee *et al*. [20] described the NHIS-NSC cohort profile in detail, including the composition of and measurements provided by the cohort, as well as ways to approach the data. Among them, Rim *et al*. [19] showed that retinal vein occlusion was associated with an increased risk of stroke, especially ischemic stroke.

Data from the Korean National Institute of Environmental Research were provided to the general public in December 2005 to satisfy public interest in air pollution. Studies using those data have shown associations between air pollution and several diseases, including respiratory and cardiovascular diseases [15, 27], stroke, and suicide [17, 28]. Kang *et al*. [15] showed that ambient air pollution, especially PM_2.5_, increased the risk of out-of-hospital cardiac arrest. Kim *et al*. [17] reported that increased levels of O_3_ and PM affect the suicide rate.

Many pathophysiological mechanisms have been suggested to explain the association between air pollutants and the incidence of OM. First, pollutants might directly induce mucosal swelling in the Eustachian tubes, causing stenosis [29]. Second, air pollutants may induce adenoidal hyperplasia, again resulting in Eustachian tube stenosis. Finally, pollutants may interfere with mucociliary clearance, which can also give rise to Eustachian tube dysfunction [30] and greater susceptibility to upper respiratory viral illnesses [31]. Several *in vivo* and *in vitro* experimental studies have demonstrated that diesel fumes and particulate matter can induce middle ear inflammation [32, 33].

We sought to improve the accuracy of data on the incidence of OM in several regards. First, we selected data from hospitals where otorhinolaryngologists and pediatricians were present to improve the accuracy of the diagnosis of OM. Proper examination of the tympanic membrane requires expert skills and ability. Thus, we excluded data where the diagnoses were made by other specialists. Second, we corrected for confounding factors that affect the incidence of OM, especially seasonal effects. The prevalence of OM is lower in the summer than in other seasons, likely because of the much lower incidence of respiratory viral diseases in the summer [7]. Third, to our knowledge, this is the first reported nationwide study to show significant relationships between air pollutants and the incidence of OM.

This study has some limitations. First, we evaluated the effects of air pollutant on OM based on weeklong lags. For example, OM occurring 6 days after exposure to an air pollutant would be considered to occur at lag 0, whereas OM occurring just 2 days later (i.e., 8 days after exposure) would be considered lag 1. That is, it could be ascribed to the discrepancy at 1 week, although it actually differs by 2 days [17]. Moreover, OM onset early in the week could be caused by air pollutants generated in the previous week, and the effect of air pollution on OM might be underestimated by using weekly averaged concentrations. In addition, we did not include upper respiratory infections despite their effect on OM because there was no information on such infections in the KNHIS [34]. To interpret the term lag response properly, data on upper respiratory diseases are required. For these reasons, we propose this is a simple hypothesis that requires further study.

Second, we did not consider the areas according to the residences of the children. We firmly believe that this method would enable drawing of a solid conclusion; thus, we will consider it for a future study. Third, the relationships between air pollutants and OM were not analyzed based on prevalence. The NHIS-NSC data comprise claims from hospitals to NHIS based on patient visits to hospitals. For example, claims for a visit to a hospital in one week and another claim the next week would not be processed together, although the real incidence of OM was one patient. To improve the incidence accuracy, we did filter repeated claims within the same week. Fourth, we considered age effects on OM incidence using age categories <5 years and ≥5 years, although the prevalence and the progress of OM in neonates and in children <2 years and those ≥2 years differ greatly. This was unavoidable because the raw data from NHIS-NSC sorted children into groups of <5 years and ≥5 years in age. Fifth, we could not consider indoor environmental factors, such as indoor renovations or new furniture, and meteorological factors, especially temperature and humidity, although they might be associated with childhood ear infections [25, 26]. Sixth, we did not consider adequate temporal and spatial resolution to obtain significant epidemiological conclusions. For temporal resolution, the effect of air pollution on OM might be underestimated by using weekly averaged air pollutants concentrations. Regarding to spatial resolution, we used the mean air pollutant concentrations in each of 16 administrative regions to evaluate the impact of air pollution on OM. The median Pearson correlations of air pollutant concentrations at different monitoring stations within the same administrative region showed high correlations at most stations but negative or low correlations at some stations. Accordingly, further studies are needed to evaluate the effect of air pollution on OM in the general population on the basis of daily and region-specific air pollution data.

In conclusion, the incidence of OM was increased immediately following and for 1–4 weeks after exposure to PM_10_, NO_2_, O_3_, SO_2_, and CO. These findings enhance our understanding of the relationships between OM and air pollutants.

## Supporting information

S1 Fig Tendencies in weekly air pollutant levels in South Korea across the year. (A) particulate matter 10, (B) nitrogen dioxide, (C) ozone, (D) sulfur dioxide, and (E) carbon monoxide
(TIF)

S1 Table Weekly otitis media incidences according to the particulate matter ≤10 μm in diameter, nitrogen dioxide, ozone, sulfur dioxide, and carbon monoxide for years and seasons in South Korea. (XLSX)

S2 Table Weekly otitis media incidences according to the particulate matter ≤10 μm in diameter, nitrogen dioxide, ozone, sulfur dioxide, and carbon monoxide for 16 administrative regions in South Korea. (XLSX)
