# Supplementary figures and images for: Air pollution influences the incidence of otitis media in children: A national population-based study
Source: PLoS One. 2018 Jun 28;13(6):e0199296. doi: 10.1371/journal.pone.0199296 (PMC6023207; doi:10.1371/journal.pone.0199296)

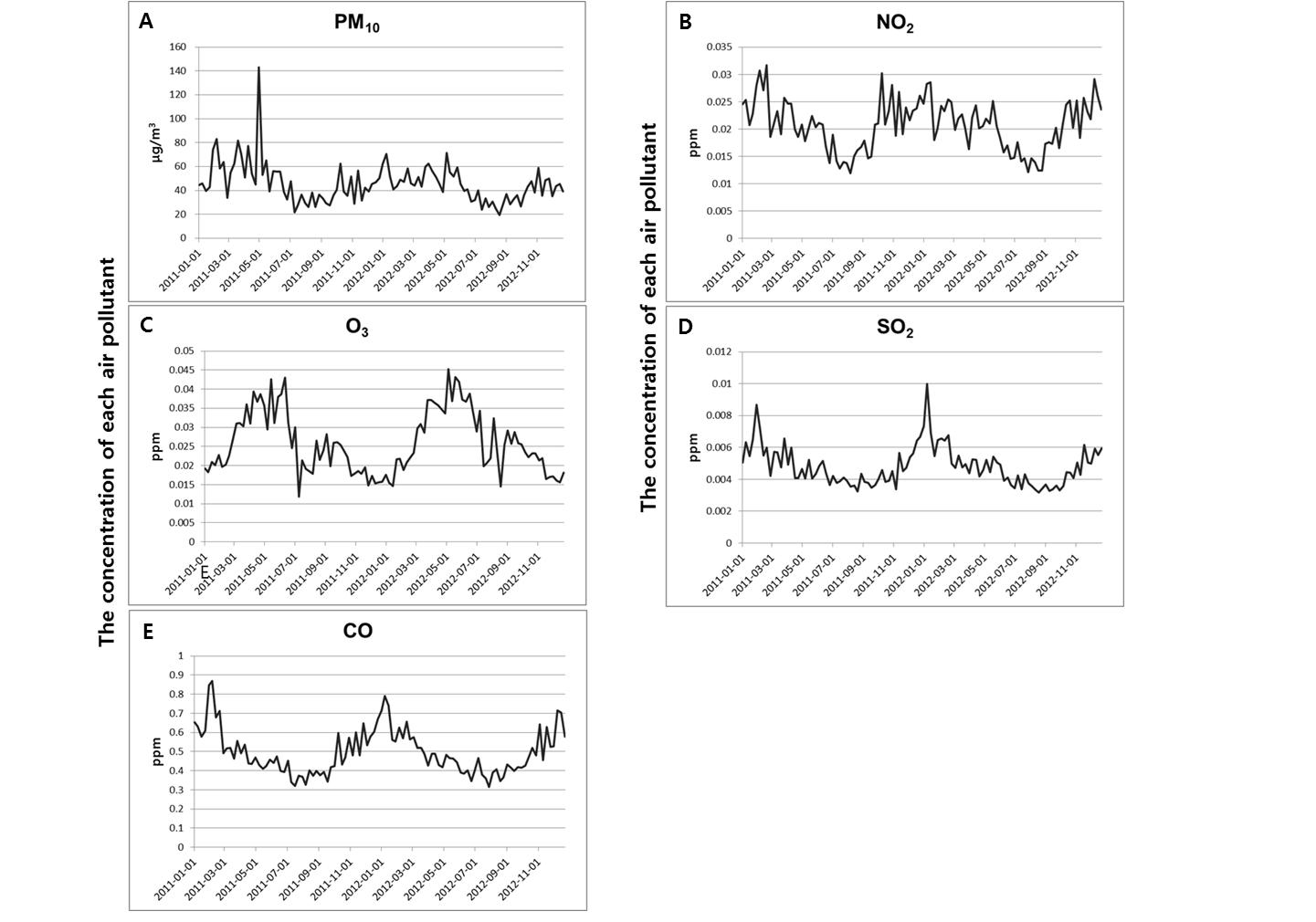

Supplement: S1 Fig — (A) particulate matter 10, (B) nitrogen dioxide, (C) ozone, (D) sulfur dioxide, and (E) carbon monoxide (TIF) [file pone.0199296.s001.tif]
